# *Poria cocos* Attenuated DSS-Induced Ulcerative Colitis via NF-κB Signaling Pathway and Regulating Gut Microbiota

**DOI:** 10.3390/molecules29092154

**Published:** 2024-05-06

**Authors:** Xiaojun Song, Wei Wang, Li Liu, Zitong Zhao, Xuebin Shen, Lingyun Zhou, Yuanxiang Zhang, Daiyin Peng, Sihui Nian

**Affiliations:** 1 School of Pharmacy, Wannan Medical College, Wuhu 241002, China; tsongxj@wnmc.edu.cn (X.S.); 20209071@stu.wnmc.edu.cn (W.W.); liulilili@stu.wnmc.edu.cn (L.L.); 20209092@stu.wnmc.edu.cn (Z.Z.); sxbchn@163.com (X.S.);; 2 School of Pharmacy, Anhui University of Chinese Medicine, Hefei 230012, China; 3 Anhui Province Key Laboratory of Chinese Medicinal Formula, Hefei 230012, China; 4 Xin’an Medicine, Key Laboratory of Chinese Ministry of Education, Anhui University of Chinese Medicine, Hefei 230038, China; 5 Anhui Provincial Engineering Laboratory for Screening and Re-Evaluation of Active Compounds of Herbal Medicines in Southern Anhui, Wannan Medical College, Wuhu 241002, China; 6 Institute of Modern Chinese Medicine, Wannan Medical College, Wuhu 241002, China; 7 Center for Xin’an Medicine and Modernization of Traditional Chinese Medicine of IHM, Wannan Medical College, Wuhu 241002, China

**Keywords:** *Poria cocos*, ulcerative colitis, gut microbiota, intestinal barrier function

## Abstract

Ulcerative colitis (UC), as a chronic inflammatory disease, presents a global public health threat. However, the mechanism of *Poria cocos* (PC) in treating UC remains unclear. Here, LC-MS/MS was carried out to identify the components of PC. The protective effect of PC against UC was evaluated by disease activity index (DAI), colon length and histological analysis in dextran sulfate sodium (DSS)-induced UC mice. ELISA, qPCR, and Western blot tests were conducted to assess the inflammatory state. Western blotting and immunohistochemistry techniques were employed to evaluate the expression of tight junction proteins. The sequencing of 16S rRNA was utilized for the analysis of gut microbiota regulation. The results showed that a total of fifty-two nutrients and active components were identified in PC. After treatment, PC significantly alleviated UC-associated symptoms including body weight loss, shortened colon, an increase in DAI score, histopathologic lesions. PC also reduced the levels of inflammatory cytokines TNF-α, IL-6, and IL-1β, as evidenced by the suppressed NF-κB pathway, restored the tight junction proteins ZO-1 and Claudin-1 in the colon, and promoted the diversity and abundance of beneficial gut microbiota. Collectively, these findings suggest that PC ameliorates colitis symptoms through the reduction in NF-κB signaling activation to mitigate inflammatory damage, thus repairing the intestinal barrier, and regulating the gut microbiota.

## 1. Introduction

Ulcerative colitis (UC) is a non-specific, chronic inflammatory bowel disease affecting the colon and rectum, characterized by erosive lesions in the mucosa and submucosa, accompanied by diarrhea, hematochezia, abdominal pain, weight loss, and other single or mixed symptoms [1]. In recent years, the incidence of UC has been relatively high in developed countries and is rapidly increasing in developing countries, posing a significant public health challenge due to its potential progression into colorectal cancer [2]. Currently, the treatment for UC is based on disease severity and includes medication therapy with amino salicylic acid, corticosteroids, immunosuppressants, TNF-α antagonists, and in severe cases, colectomy [3,4]. However, the high economic burden and adverse reactions significantly impact the patients’ quality of life and impose a considerable financial burden on both patients and society [5]. Therefore, numerous potential drugs have undergone extensive investigation to attain a safe and effective therapy for UC.

Currently, the cause of UC remains incompletely understood. However, the pathogenesis of UC development is widely acknowledged to be influenced by genetic factors, immune dysfunction, gut flora changes, and environmental influences [6,7,8]. The hallmark feature of UC is the persistent and intense inflammation caused by immune cell infiltration into the intestinal mucosa. The first response of cells to harmful stimuli is to recruit neutrophils to augment the inflammatory cascade and release other pro-inflammatory cytokines and chemokines (such as TNF-α and IL-6) [9,10]. The downregulation of the NF-κB pathway has been demonstrated to reduce the levels of IL-1β and TNF-α and IL-6 in the colon, thereby ameliorating DSS-induced inflammation [11]. Moreover, evidence suggests that patients with UC exhibit significant alterations in their intestinal microbiome, including reduced richness and diversity [12,13]. Such disturbances may contribute to the development of overactive intestinal inflammation or immune overreaction. Therefore, targeting the downregulation of NF-κB and regulating gut microbiota homeostasis using potential therapeutic agents may present promising anti-inflammatory strategies for managing UC. Patients with colitis consistently exhibit reduced expression of tight junction proteins in the intestinal epithelium, such as ZO-1 and Claudin-1, and increased epithelial permeability [14]. These changes ultimately lead to the onset and recurrence of UC. In this regard, preserving the integrity of the intestinal mucosa, eliminating pathogenic microorganisms, modulating the host’s immune system, and maintaining gut microbiota homeostasis play a crucial role in the treatment of UC.

*Poria cocos* (Schw.) Wolf (PC), a traditional Chinese medicine (TCM) with medicinal and culinary applications, is widely available in China [15,16]. In the theoretical framework of TCM, UC is classified as a type of diarrhea, dysentery, and hematochezia based on its clinical characteristics. PC has been widely employed for treating diarrhea in China [17]. Modern research has demonstrated that the main bioactive components of PC including polysaccharides and triterpenes have dormitive, anti-tumor, anti-inflammation, anti-oxidation, and anti-diabetic effects [18,19,20,21,22]. The aforementioned research indicates that PC holds promising potential for the management of UC symptoms including inflammation and diarrhea. However, there is currently a lack of research on the mechanism of PC in treating UC.

In this study, we evaluated the potential of PC extract (PCE) for the treatment of colitis in mice. Dextran sulfate sodium (DSS)-induced UC mice were used to investigate the potential ameliorative effects of PCE by investigating its effects on the histological changes, inflammatory cytokines, molecular mechanisms of anti-inflammation, intestinal barrier function, and the alterations in the intestinal bacterial community structure. Moreover, the chemical profile of PC was characterized by LC-MS. This work sheds new light on the potential application of PC as a promising therapeutic agent for UC and contributes to our understanding of its pharmacological mechanisms.

## 2. Results

### 2.1. Chemical Profile Analysis and Determination of Polysaccharide of PC

The chemical profiling of PC was characterized by UPLC-Q-Exactive-MS in positive and negative ion modes (Supplementary Figure S1). We compared PC with the reference substances database (LuMet-TCM) for compound identification, based on retention time, relative molecular mass, MS fragmentations, and molecular formulae. The identification strategy includes three conditions: (1) the error between the retention time and the standard retention time in the database falls within ±0.2 min; (2) the first-order molecular weight error is limited to 5 ppm; (3) a comparison is made between the measured MS^2^ and the standard MS^2^. As a result, 52 compounds were identified or tentatively identified in PCE, including triterpenes, amino acids, and phenolics (Supplementary Table S1). In addition, the main polysaccharide content in the water extraction of *Poria cocos* was determined by the anthrone–sulfuric acid method [19], and the polysaccharide content reached 80% (Supplementary Tables S2 and S3).

### 2.2. PCE Alleviates the Symptoms of DSS-Induced Ulcerative Colitis in Mice

**Figure 1:**
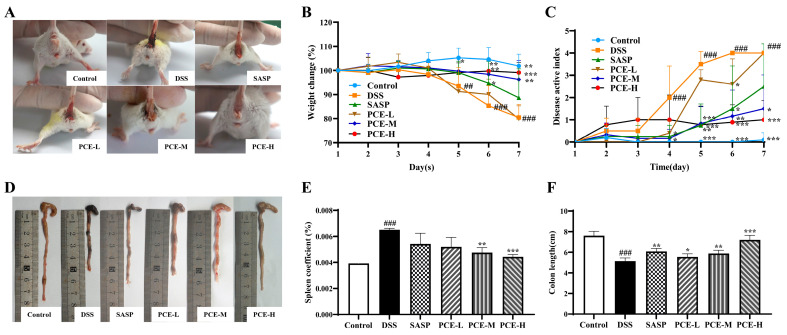
PCE relieves DSS-induced colitis in mice. (**A**) Perianal condition in mice; (**B**) weight change during the experiment; (**C**) disease activity index (DAI) score; (**D**,**E**) representative images of colon length on d7; (**F**) spleen coefficients. PCE-L, low dose of PCE; PCE-M, medium dose of PCE; PCE-H, high dose of PCE. The values are expressed as mean ± SD (*n* = ten mice in each group) ^##^ *p* < 0.01, ^###^ *p* < 0.001 vs. control group; * *p* < 0.05, ** *p* < 0.01, *** *p* < 0.001 vs. model group.

**Figure 2:**
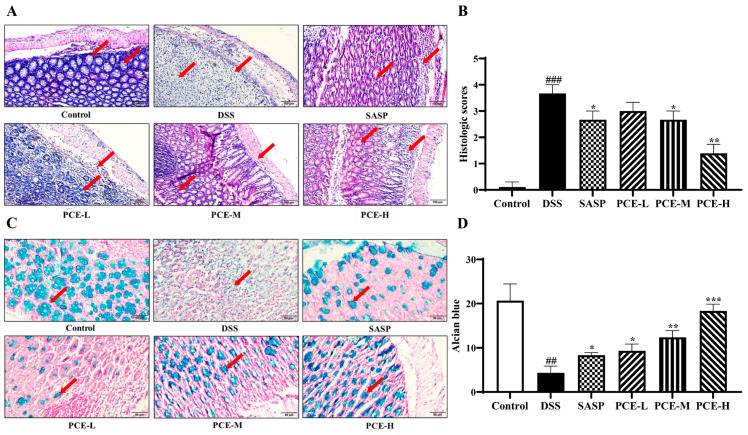
PCE ameliorates DSS-induced intestinal structural damage in UC mice. (**A**) Representative H&E staining of colon tissue (200×); (**B**) histological score of H&E staining; (**C**) Alician staining of goblet cells (400×); (**D**) histological score of Alician staining. Red arrow indicates inflammatory damage before and after administration in the blank group and the model group. The values are expressed as mean ± SD (*n* = 3). ^##^ *p* < 0.01, ^###^ *p* < 0.001 vs. control group; * *p* < 0.05, ** *p* < 0.01, *** *p* < 0.001 vs. model group.

To investigate the effect of PC on UC, the male Balb/c mice were subjected to a 7-day treatment with 3.0% DSS to establish an acute colitis model that exhibited clinical symptoms closely resembling those observed in human colitis, including body weight loss, diarrhea, and rectal bleeding. Meanwhile, UC mice received a daily oral administration of PCE (low, medium, and high doses, and recorded in Section 4.5) and sulfasalazine (SASP) (200 mg/kg) for seven days. In the PCE groups and the SASP group, the perianal prolapse and hematoma in mice were significantly improved compared to the DSS group (Figure 1A). Moreover, a notable reduction in weight loss was observed in the mice treated with medium and high doses in the PCE groups, as well as in the SASP group (Figure 1B). The Disease Activity Index (DAI) score, a reliable indicator of colitis severity, showed a significant increase from the fourth day onwards. However, treatment with medium and high doses in the PCE groups and the SASP group treatment effectively reversed this elevation (Figure 1C). DSS-induced splenomegaly and colonic shortening were improved by the treatment, especially after treatment with medium and high doses in the PCE groups and SASP group treatment (Figure 1D–F). Furthermore, histological analysis using H&E staining revealed that the treatment in the PCE groups effectively ameliorated the DSS-induced damage in both the muscular layers and crypts of the colon tissue in a dose-dependent manner. Additionally, these groups exhibited a reduction in inflammatory cell infiltration (Figure 2A,B). Alician staining was employed to detect mucus secretion in the colon tissue. Compared with the control group, DSS significantly reduced mucus secretion, while PCE improved it (Figure 2C,D). Overall, these results indicated that PC effectively mitigated DSS-induced colitis.

### 2.3. PCE Alleviates Colonic Inflammation in DSS-Induced UC Mice

**Figure 3:**
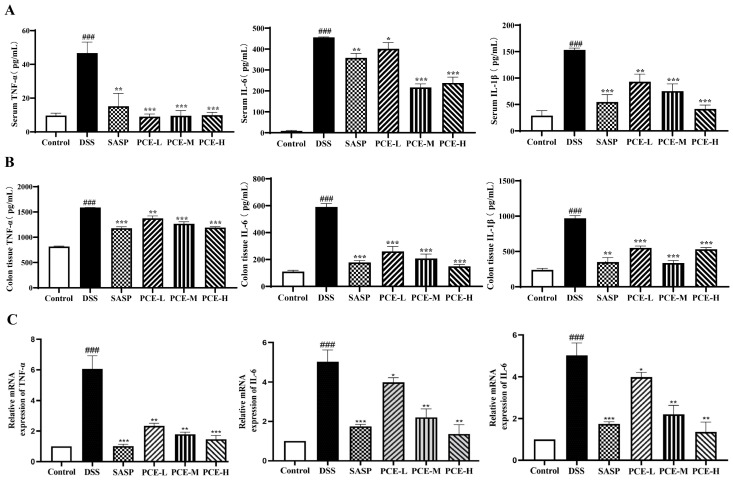
PCE decreased inflammatory cytokine levels in serum and colon tissue. (**A**,**B**) The expressions of TNF-α, IL-6, and IL-1β levels in serum and colonic tissue revealed by ELISA, respectively; (**C**) the mRNA expressions of TNF-α, IL-1β, and IL-6 were detected by performing qPCR. The values are expressed as the means ± SD (*n* = 3 of independent experiments in vitro), ^###^ *p* < 0.001 vs. control group; * *p* < 0.05, ** *p* < 0.01, and *** *p* < 0.001 vs. DSS group.

As inflammatory cytokines are key factors in the onset of colitis, we assessed the impact of PCE on proinflammatory cytokine levels. We measured the inflammatory cytokines in the mouse serum and colon tissue using ELISA. As shown in Figure 3A,B, the levels of IL-1β, TNF-α, and IL-6 in the serum and colons of mice treated with DSS exhibited a significant increase compared with the control group. Conversely, the UC mice treated with the low, medium, and high doses of PCE, as well as SASP showed a significant reduction in the levels of IL-1β, TNF-α, and IL-6 in serum and colon tissue compared to the DSS-treated mice. Moreover, we also observed a significant downregulation of TNF-α, IL-6 and IL-1β mRNA levels in colon tissue treated with PCE (Figure 3C).

**Figure 4:**
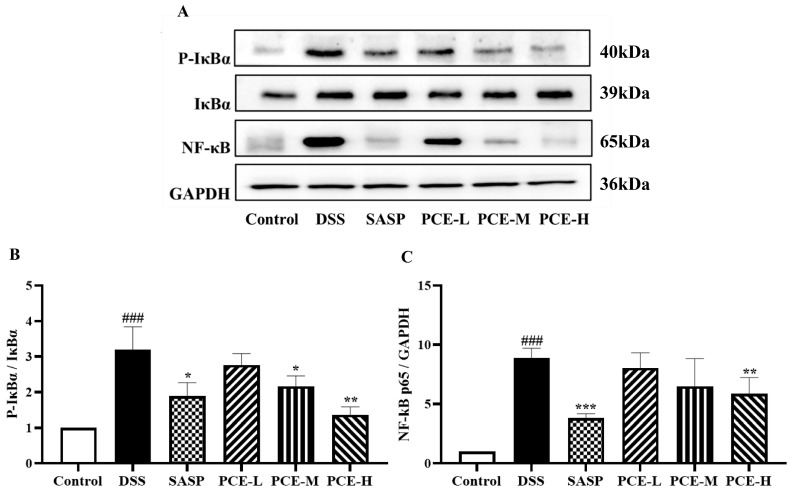
PCE inhibits ΙκΒα/ΝF-κΒ activity. (**A**) Immunoblots of ΙκΒα, P-ΙκΒα, and iNOS in the colon tissue of mice; (**B**,**C**) protein expression chart. The values are expressed as mean ± SD (*n* = 3 of independent experiments in vitro), ^###^ *p* < 0.001 vs. control group; * *p* < 0.05, ** *p* < 0.01, *** *p* < 0.001 vs. DSS group.

Furthermore, our findings demonstrate that PCE exerts an inhibitory effect on the NF-κB signaling pathway, thereby exhibiting potent anti-inflammatory properties. As shown in Figure 4A, DSS-induced UC mice exhibited a significant upregulation of the protein expression levels of IκBα, P-IκBα, and NF-κB in colon tissue. However, treatment with PCE at the high dose significantly decreased UC-induced expression of IκBα, P-IκBα, and NF-κB compared to the control group (Figure 4A–C and Figure S2). These results suggest that the administration of PCE effectively attenuated the inflammatory response in mice with DSS-induced colitis.

### 2.4. PCE Ameliorates Intestinal Barrier Function in DSS-Induced UC Mice

**Figure 5:**
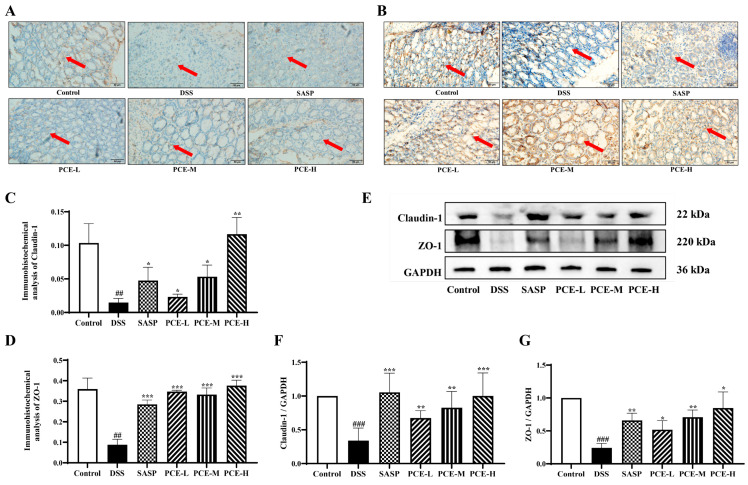
PCE attenuates DSS-induced damage to the TJs of intestinal epithelium in mice. (**A**–**D**) TJ proteins Claudin-1 and ZO-1 were observed by immunohistochemistry; (**E**–**G**) the expression of ZO-1, Claudin-1 proteins were determined by Western blotting. Red arrow indicates TJs damage before and after administration in the blank group and the model group. The values are expressed as mean ± SD (*n* = 3 of independent experiments in vitro), ^##^ *p* < 0.01, ^###^ *p* < 0.001 vs. control group; * *p* < 0.05, ** *p* < 0.01, *** *p* < 0.001 vs. DSS group.

Intestinal barrier damage is a prominent pathological feature observed in both DSS-induced animal models and UC patients. Consequently, we investigate whether PCE remodeled the structure and function of the intestinal barrier in DSS-induced UC mice. Intestinal epithelial tight junctions (such as ZO-1 and Claudin-1) among the intestinal epithelial cells are crucial constituents of the intestinal epithelial barrier, impeding the infiltration of environmental toxins, luminal antigens, and bacteria to prevent potential focal enteropathy or systemic disease. As expected, immunohistochemistry analysis showed that the mice treated with DSS showed severe damage to the colon epithelium, and after treatment with PCE, the PCE groups showed significant improvement to the integrity of the intestinal epithelium (Figure 5A,B). Furthermore, PCE effectively restored the downregulation expression of ZO-1 and Claudin-1 in DSS-induced UC mice (Figure 5E–G and Figure S2). Collectively, these findings suggest that PCE could protect intestinal barrier function in UC mice.

### 2.5. PCE Regulates Gut Microbiota Disorders in DSS-Induced UC Mice

**Figure 6:**
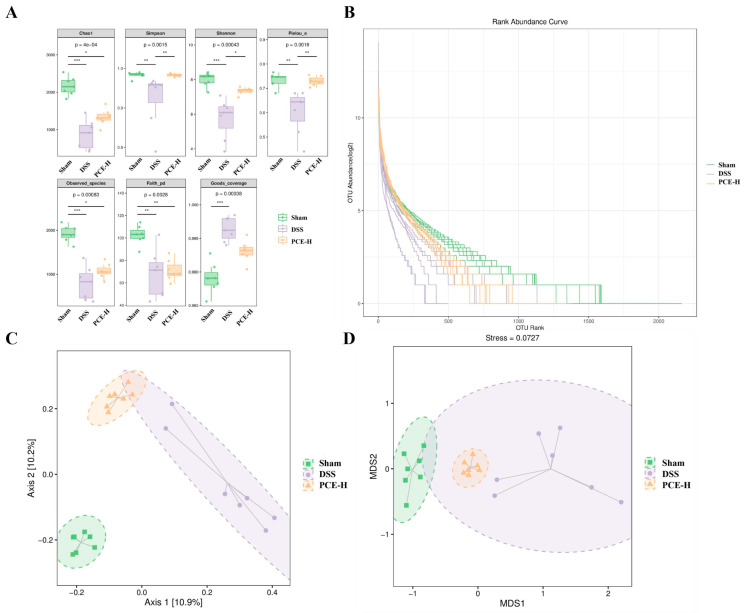
Intestinal flora analyses in mice. (**A**) Alpha diversity; (**B**) abundance curve; (**C**) principal coordinates analysis; (**D**) non-metric multidimensional scaling. The values are expressed as mean ± SD. * *p* < 0.05, ** *p* < 0.01, and *** *p* < 0.001 vs. DSS group.

The impact of PCE on gut microbiota composition was investigated using 16S rRNA gene sequencing of the cecal contents from DSS-induced ulcerative colitis mice. Our results showed that the DSS treatment significantly reduced both Chao1 and the observed species’ alpha diversity indices in mice, compared to the control group, indicating a decrease in microbiota diversity induced by DSS (Figure 6A). The higher the Shannon and Simpson indices, the higher the community diversity; PCE-H enhanced these two indices in DSS-treated mice. The evenness of the abundance grade curve reflects the evenness of the community composition (Figure 6B). The microbial community composition was examined using PCoA analysis to investigate similarities or differences. Notably, the gut microbiota of the DSS group exhibited distinct separation from that of the control group (Figure 6C), indicating an induced imbalance in the intestinal flora due to DSS treatment. Interestingly, the PCE-H treatment groups displayed a closer resemblance to the control group than to the DSS group, suggesting a protective effect of PCE against DSS-induced dysbiosis. Relevantly, comparable outcomes were obtained in the NMDS analysis (Figure 6D).

**Figure 7:**
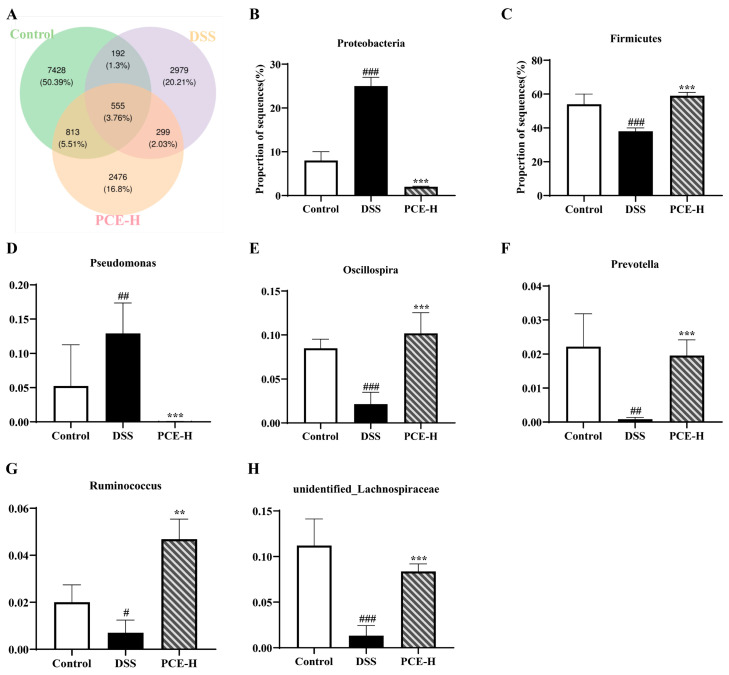
Intestinal microbiota species composition changes in mice. (**A**) Venn diagram; (**B**–**H**) relative abundance comparisons between *Proteobacteria*, *Firmicutes*, *Pseudomonas*, *Oscillospira*, *Prevotella*, *Ruminococcus*, and unidentified-*Lachnospiraceae* in three groups, respectively. ^#^ *p* < 0.05, ^##^ *p* < 0.01, and ^###^ *p* < 0.001 vs. control group; The values are expressed as mean ± SD, ** *p* < 0.01, *** *p* < 0.001 vs. DSS group.

Subsequently, we conducted a taxonomic analysis of the bacterial communities at the phylum and genus levels. Furthermore, we performed a significant difference test between the groups based on the abundance data to assess the statistical significance of species abundance variations. As shown in Figure 7A, the three groups shared a total of 555 species, accounting for 3.76% of the overall composition. Among these, the control group exhibited 7428 unique species, representing 50.39% of the entire dataset, while the DSS and PCE-H groups displayed 2979 (20.21%) and 2476 (16.8%) unique species, respectively. At the phylum level, the DSS group exhibited a significant increase in Proteobacteria abundance and a decrease in Firmicutes abundance compared to the control group. In contrast, the PCE-H group reversed the dysbiosis induced by DSS treatment (Figure 7B,C). At the genus level, compared to the control group, we observed a significant increase in Pseudomonas abundance and a significant decrease in *Oscillospira*, *Prevotella*, *Ruminococcus*, and unidentified-*Lachnospiraceae* abundance in the DSS group. Importantly, administration of PCE-H effectively reversed these alterations induced by DSS (Figure 7D–H).

## 3. Discussion

Ulcerative colitis is a chronic and recurrent autoimmune disorder characterized by the disruption of mucosal barrier integrity, alteration of gut microbiota composition, and impairment of immune function [23,24]. In this study, we reported that DSS-induced UC mice treated with PCE experienced significant improvements to symptoms of diarrhea, bloody stools, body weight loss, colonic atrophy, intestinal epithelial destruction, and intestinal barrier dysfunction in colon tissues. Overall, our studies indicated that PC may be a potential agent for the treatment of UC.

PC is commonly found in the market as a form of nourishment, food or supplementary medication, such as porridge, soup, tea and alcoholic beverage, and these products serve to supplement and regulate the body’s nutritional needs [25]. The triterpenoids in PC have been shown to enhance non-specific immunity by stimulating the secretion of interferon γ [26]. Furthermore, recent studies have illustrated that polysaccharides are also major classes of secondary metabolites in PC. In our work, the chemical profiling of PC was identified by UPLC-Q-Exactive-MS. A total of 52 compounds were identified or tentatively characterized in PCE, including triterpenes, amino acids, phenolics and the content of polysaccharide in the water extraction reached 80%. Remarkably, the PC polysaccharides exhibit a wide range of biological activities, including antitumor, anti-inflammatory, antioxidant, immunomodulatory, hepatoprotective, and modulation on gut microbiota [27]. However, the effectiveness of *Poria cocos* polysaccharides, triterpenoids, or their combination in the treatment of UC remains uncertain. Therefore, we would like to uncover the pharmacodynamic substance of *Poria cocos* in the treatment of colitis and its potential mechanism of action.

Cumulative investigations have consistently demonstrated that impaired intestinal epithelial barrier function, involving immune, mechanical, chemical, and biological defenses, plays a crucial role in the development of UC [28,29]. The normal intestinal epithelium selectively absorbs nutrients and effectively prevents the invasion of harmful components. The colon length was significantly reduced in UC mice [7,30]. In the study, the administration of PCE significantly ameliorated weight loss, diarrhea, hematochezia, and colonic length of UC mice. Histological analysis indicated that the administration of PCE effectively ameliorated DSS-induced damage in the muscular layers and crypts of the colon tissue, while also reducing mucus secretion in the colon tissue. Moreover, the disruption of the intestinal epithelial barrier can lead to the increased permeability of the intestinal epithelium, which is closely associated with the composition of the apical junction complex comprising tight junctions (TJs) and adherent junctions (AJs) [31]. The TJs, especially, play a decisive role in serving as constitutive barriers for epithelial cells. The TJ is composed of a diverse array of proteins, including claudin, occludin, tricellulin, and junctional adhesion molecule-A (JAM-A), such as zonula occludins (ZO) and cingulin, which localize near the apical region of the epithelial cell membrane [32,33], and the downregulation of intestinal epithelial TJ proteins ZO-1 and Claudin-1 has been confirmed in patients with UC or colitic mice. Therefore, we further demonstrated the restoration of TJ proteins expression by PC through immunohistochemistry and Western blot analysis, indicating PC effectively restored the integrity of the intestinal epithelium by reinstating the TJs, thereby reducing intestinal permeability and restoring the functionality of the intestinal epithelial barrier.

The transcription factor NF-κB serves as the pivotal regulator of immune responses, and its persistent activation is closely associated with inflammatory conditions such as UC [34]. As the integrity of the barrier is compromised, there is an increase in intestinal permeability, allowing inflammatory molecules and luminal bacteria to access various organs. These harmful cellular stimuli can activate the NF-κB pathway in intestinal epithelial cells, leading to transcription and the production of proinflammatory cytokines TNF-α, IL-1β, and IL-6. In addition, these cytokines can directly exacerbate TJ protein expression and distribution [10,35]. For instance, elevated levels of TNF-α can disrupt the structure and function of the TJs [36], while IL-1β could increase TJ permeability in human intestinal epithelial cells [37]. We further demonstrated that elevated cytokine levels or decreased TJs were rescued by the administration of PCE. Furthermore, the results of Western blotting indicated a decrease in the expression of IκBα and NF-κB p65 following PCE administration, suggesting that the ameliorative effect of PCE on UC may be attributed to its regulation of the IκBα/NF-κB signaling pathway.

The gut microbiota is a complex and diverse community that establishes a superorganism with its host, regulating the host’s physiology. A healthy microbiota provides numerous benefits to the host, including improved intestinal epithelial barrier function, balanced immune systems, and essential metabolites. Unfortunately, dysbiosis of the gut microbiota is associated with various pathological conditions such as UC [38,39]. Relevant symptoms of ulcerative colitis (UC) were observed in our DSS mouse model, accompanied by a decrease in gut microbiota diversity and enrichment. Markedly, the administration of PCE-H successfully restored the aforementioned diversity and enrichment. *Firmicutes* enrichment is consistently observed in patients with ulcerative colitis and/or animal models. *Proteobacteria*, which include pathogenic bacteria such as *Salmonella*, *Vibrio cholerae*, *Escherichia coli*, *Helicobacter pylori* and other species, are also represented [40,41]. DSS treatment induced an increase in Proteobacteria levels; however, this abundance was reduced by PCE treatment, consistent with findings from other natural plant studies [42].

At the genus level, DSS disrupted the abundance of *Oscillospira*, *Prevotella*, *Pseudomonas*, *Ruminococcus* and *Lachnospiraceae*. However, this phenomenon was ameliorated by PCE administration. *Oscillospira* is a spirillum genus that has been linked to obesity, emaciation, gallstones, and chronic constipation. It also exhibits some correlations with positive or negative changes in the course of UC. Bacteria that produce short-chain fatty acids such as butyrate are promising candidates for next-generation probiotics [43]. Prevalent bacteria are typically associated with a healthy plant-based diet and play a probiotic role in the body. Their depletion has been linked to certain diseases, such as intestinal inflammation and psychiatric disorders [44]. *Pseudomonas* is widely distributed throughout nature, including on human skin, within the intestines, and the respiratory tract; it is also a highly prevalent opportunistic pathogen in clinical settings [45]. *Ruminococcus* plays a crucial role in the digestion of resistant starch, and its decreased abundance has been linked to various intestinal, immune, and nervous system disorders [46]. *Lachnospiraceae* is a member of the *Firmicutes* family that commonly inhabits the intestines of healthy individuals and may have potential benefits in regulating conditions such as asthma, gastric cancer, and type 2 diabetes, among others. Fortunately, our UC mouse model showed significant increases in *Oscillospira*, *Prevotella*, *Ruminococcus* and *Lachnospiraceae* after PCE administration [47].

## 4. Materials and Methods

### 4.1. Plant Materials and Reagents

The plant was procured from Bozhou market of traditional Chinese medicine in Anhui province and authenticated as *Poria cocos* (Schw.) Wolf by Prof. Sihui Nian (Wannan Medical College) based on Chinese Pharmacopeia (Part I, 2020 edition). The voucher specimen (No. AHWNMCSOP-22-20) has been deposited at the College of Pharmacy, Wannan Medical College in Wuhu, China.

Dextran sulfate sodium (DSS, 36–50 KDa) was purchased from MP Biomedicals (Ontario, USA). 5-Aminosalicylic acid (5-ASA) was purchased from Shanghai Xinyi Tianping Pharmaceutical Co., Ltd. (Shanghai, China). Hematoxylin and eosin (H&E) solution, Alicia acidizing solution were purchased from Biotechnology Co. Ltd. (Dalian, China). Primary antibodies (Claudin-1, ZO-1, anti–NF–κB, anti-phospho–NF–κB, anti-IκB, and anti-phospho-IκB) were purchased from Cell Signaling Technology (Beverly, MA, USA) and Abcam (Cambridge, UK). Mouse ELISA kits were from ABclonal technology Co., Ltd. (Wuhan, China).

### 4.2. Preparation of Poria cocos Extraction

In order to fully extract the major triterpenes and polysaccharides of PC, 95% ethanol and pure water were utilized for the extracts, respectively. Firstly, the powder of PC was extracted three times for 2 h each time under 95% ethanol (material: ethanol ratio = 1:12) at boiling, and the combined extracts were concentrated by a rotary evaporation unit (Shanghai Ailang Instrument Co., Ltd., Shanghai, China) to afford PC ethanol extracts (PCEE). Then, the residue of PC was dried and diluted in distilled water (material: liquid ratio = 1:12) for extraction three times at boiling for 2 h each time; the extracting solution was treated under reduced pressure to obtain PC water extracts (PCWE). Finally, PCE was obtained by mixing PCEE and PCWE.

### 4.3. UPLC-Q-Exactive Orbitrap MS/MS Analysis and Determination of Polysaccharides from Poria cocos Extraction

#### 4.3.1. Instrumentation and UPLC-Q-Exactive Orbitrap MS Conditions

UPLC-Q-Exactive Orbitrap MS/MS analysis was performed by a liquid chromatography–mass spectrometry system composed of ACQUITY UPLC I-Class plus with Waters ACQUITY UPLC HSS T3 (100 mm × 2.1 mm, 1.8 μm; Waters Technologies, Milford, MA, USA) tandem Q-Exactive Orbitrap mass spectrometer (Thermo Fisher Scientific, Waltham, MA, USA) equipped with an electron spray ionization (ESI) source. The mobile phase of chromatographic separation consisted of A (0.1% formic acid in water) and B (acetonitrile), using a gradient elution of 5% B at 0–2 min, 5–30% B at 2–4 min, 30–50% B at 4–8 min, 50–80% B at 8–10 min, 80–100% B at 10–14 min, 100% B at 14–15 min. The flow rate was 0.35 mL/min and the column temperature was held at 45 °C. The sample injection volume was 5 μL. The ESI source was operated to acquire MS spectra by full scan from *m*/*z* 100 to 1200 at a resolution of 70,000. Other parameters were set as follows: full MS/dd-MS^2^ mode, capillary temperature, 320 °C; ion source temperature, 350 °C; sheath gas flow rate, 35 arb; aux gas flow rate, 8 arb; electrospray voltage, 3.8 kV (ESI+) or 3.0 kV (ESI−); normalized collision energy, 10%, 30%, and 40%. An external calibration for mass accuracy was carried out before analysis.

Then, the PCE was dissolved in 95% ethanol–aqueous solution with a sample concentration of 10 mg/mL for UPLC-Q-Exactive Orbitrap MS analysis. The content of polysaccharide in the PC water extracts was determined by anthrone–sulfate colorimetry method [19].

#### 4.3.2. MS Data Preprocessing and Statistical Analysis

The original LC-MS data were processed using Progenesis QI V3.0 software (Nonlinear Dynamics, Newcastle, UK) for baseline filtering, peak identification, integration, retention time correction, peak alignment, and normalization. The main parameters used were a precursor tolerance of 5 ppm and a product tolerance of 10 ppm. The identification of the compounds was based on matching the molecular formulas, retention times, and MS fragmentation patterns with corresponding standard substances, references, and online databases involving LuMet-TCM (Luming Biotech CO., Ltd., Shanghai, China).

The qualitative substances identified in the QI search database are retained as the original ingredient if they have a total score exceeding 50 points, or a total score surpassing 40 points along with a secondary matching score greater than 50 points.

### 4.4. Animals

The male Balb/c mice (age, 6–8 weeks; weight, 18–22 g) were purchased from Henan SkBex Biotechnology (certificate no. SCXKSYXK (Wan) 2018-004), China. All the mice were housed in specific pathogen-free conditions with 24 °C ± 2 °C and 12/12 h dark/light cycles (experimental Animal Center of Wannan Medical College, Wuhu, Anhui province, China) and permitted free access to water and standard rodent food. The animal research was carried out in agreement with the Chinese legislation on laboratory animals and approved by our university Ethics Committee.

### 4.5. Establishment and Treatment of UC

The development of UC was induced by providing free access to a freshly prepared 3% DSS (W/V) solution for seven consecutive days [48]. Balb/c mice were randomly divided into six groups (*n* = 10): (1) control group (0.9% saline), (2) UC group (3% DSS solution), (3) SASP (200 mg/kg) group, (4) low PCE dose group (PCE-L, 20.46 mg/kg/d PCEE + 35.5 mg/kg/d PCWE), (5) medium PCE dose group (PCE-M, 40.92 mg/kg/d PCEE + 71.0 mg/kg/d PCWE), and (6) high PCE dose group (PCE-H, 81.84 mg/kg/d PCEE + 142.0 mg/kg/d PCWE). Mice in the control group were free to drink distilled water, mice in the other groups were free to drink 3% DSS solution (dissolved in sterile water) for 7 days. Meanwhile, the SASP group was administered 200 mg/kg/d of SPAP for seven days, and the PCE-L/M/H groups were treated with the above doses of PCE by intragastric administration for a period of seven days, respectively. Thereafter, the weight, stool consistency, and perianal status of each mouse were recorded daily to monitor the severity of disease. The DAI score was calculated according to the scoring criteria. The histological grading scheme was described as follows: weight loss percentages (WLP) = 0 if weight is unchanged, WLP = 1 if the weight change percentage is 1–5, WLP = 2 if the weight change rate is 5–10, WLP = 3 if the weight change rate is 10–15, and WLP = 4 if the weight change rate is >15; stool consistency (SC) = 0 for normal stool, SC = 2 for loose stool, and SC = 4 for diarrhea); stool bleeding (SB) = 0 for normal, positive occult blood SB = 2, and overt bleeding SB = 4. Total scores from the three indices were divided by 3 to provide the DAI = (WLP + SC + SB)/3.

After 8 days of DSS modeling, animals were anesthetized with pentobarbital sodium. Then, blood samples were collected from each group via the rat’s retro-orbital plexus and centrifuged at 3000× *g* for 10 min to obtain serum, which was subsequently stored at −80 °C. After blood collection, the mice were sacrificed by cervical dislocation. The colon of each mouse was collected and the length was measured, followed by measurement of the distance from anus to the ileocecal valve. A suitable amount of colon samples was fixed in 4% paraformaldehyde, followed by embedding and slicing procedures for further analysis. The remaining parts were stored at −80 °C for subsequent analysis.

### 4.6. Hematoxylin & Eosin Staining

Intestinal tissue was fixed in 4% paraformaldehyde, embedded in paraffin, and subsequently dewaxed according to instructions. Sections were then stained with H&E solution, dehydrated, and mounted with neutral glue. Finally, the samples were examined under a microscope for image analysis.

### 4.7. Alician Staining

The aforementioned methods were employed for the preparation of histological sections, and then dewaxed tissue sections were immersed in Alicia acidizing solution for 3 min, followed by staining with Alicia dye solution for 30 min and subsequent rinsing under running water. The sections were subsequently immersed in nuclear fast red dye solution for 5 min, followed by rinsing with running water, dehydration using a gradient ethanol series, and sealing with neutral gum after xylene treatment.

### 4.8. Enzyme-Linked Immunosorbent Assay (ELISA)

The intestinal tissues were equilibrated to ambient temperature, quickly washed in Tris-buffered saline containing 0.1% Tween-20 (TBST), and then lysed in lysis buffer on ice for 30 min. The supernatants were obtained by centrifugation at 14,000× *g* for 15 min at 4 °C. The levels of IL-1β, IL-6, and TNF-α in serum or intestinal tissue supernatants were detected by ELISA kits (ABclonal, Wuhan, China) according to the manufacturer’s instructions. The optical density value was measured at 450 nm by a microplate reader (Bio-Rad, Hercules, CA, USA).

### 4.9. Quantitative PCR (qPCR) Analysis

The mRNA expression of TNF-α, IL-6, and IL-1β in colon tissues was assessed by performing qPCR assay. Firstly, the total RNA was extracted using TRIzol reagent (R401-01, Biosharp, Hefei, China), and the concentration as well as purity were assessed using a NanoDrop. Subsequently, the RNA was prepared and the reverse transcriptase reaction was performed by using Reverse Transcription Kit (BL699A, Biosharp, China). Lastly, the obtained complementary DNA (cDNA) and corresponding primers listed in Supplementary Table S4 were used for qPCR amplification in the ThermoFisher QuantStudio 3 Real-Time PCR System. The reaction conditions were as follows: 95 °C, 2 min initial denaturation, and 40 amplification cycles (95 °C, 15 s, and 60 °C, 30 s). After the amplification, the CT values of each sample were normalized to GAPDH, and the mRNA expression levels of genes were calculated using the 2^−ΔΔCT^ method for relative quantification.

### 4.10. Immunohistochemistry Staining

Immunohistochemistry was performed following previously established protocols with some modifications implemented [23]. Briefly, 5 μm paraffin-embedded colon tissue sections were subjected to antigen retrieval by deparaffinization in xylene, dehydration in various alcohol solutions, and treatment with citrate buffer. Endogenous peroxidase was then generated by adding 3% hydrogen peroxide for 15 min. The samples were incubated overnight at 4 °C with primary antibodies against Claudin-1 or ZO-1 (1:200, Santa Cruz Biotechnology, Shanghai, China). The next day, the sections were subjected to three rounds of washing with phosphate buffered saline for 5 min each time. Subsequently, they were incubated with a corresponding secondary antibody at room temperature for 30 min. Finally, images were captured using an optical microscope.

### 4.11. Western Blotting

Colon tissue and lysates were homogenized at a ratio of 1:8, followed by centrifugation of the lysates. The resulting supernatants were mixed with a sample protection solution in a ratio of 1:4 ratio and boiled for 10 min. Proteins were separated using sodium dodecyl sulfate-polyacrylamide gel electrophoresis (6%–10%) and transferred to nitrocellulose membranes. After washing in skimmed milk (50 g/L) for 2 h at room temperature, the membranes were incubated overnight at 4 °C with primary antibodies against Claudin-1 (1:2000, sc-166338, Santa Cruz Biotechnology), ZO-1 (1:2000, sc-33725, Santa Cruz Biotechnology), P-ΙκΒα (1:2000, 2859, CST, Shanghai, China), ΙκΒα (1:2000, 4812, CST), ΝF-κΒ P65 (1:2000, 8242, CST), and GAPDH (1:2000, BL006B, Biosharp). Subsequently, the membranes were washed three times with TBST for 10 min each and then incubated with a secondary antibody at room temperature for 1 h. The enhanced chemiluminescent reagent was added to the membranes, and protein levels were quantified using Image J software 1.x.

### 4.12. Gut Microbiota Profiling by 16S rRNA Sequencing

Total microbial genomic DNA was extracted from fecal samples and the quality of the DNA extracts was determined using 1.2% agarose gel electrophoresis, and the concentration and purity of genomic DNA were quantified using a Nanodrop 2000. To amplify target fragments via polymerase chain reaction (PCR), primers were designed based on the conservative regions in the sequence. The PCR amplification of rRNA gene variable regions (single or multiple consecutive) or specific gene fragments was performed according to sample-specific barcode sequences. Amplified products were purified and recovered using magnetic beads, then quantified by fluorescence with a Quant-iT PicoGreen dsDNA Assay Kit and microplate reader (BioTe, FLx800). Based on the fluorescence quantitative results, the samples were proportionally mixed to meet the sequencing quantity requirements of each sample. Subsequently, a high-throughput sequencing library was prepared.

### 4.13. Statistical Analysis

The results of H&E, Alician, Western blotting, and immunohistochemistry were analyzed using Image J software. The data were processed using SPSS 22.0 software, and the data are presented as mean ± SD of at least three independent experiments in vitro or six mice of each group in vivo, calculated using one-way analysis of variance and Tukey’s tests. *p* < 0.05 was considered as statistical significance.

## 5. Conclusions

In conclusion, our investigation demonstrated that PC could alleviate UC symptoms, attenuate excessive inflammatory levels, repair intestinal barrier function, and restore the imbalanced gut microbiota in a DSS-induced UC mouse model. As a TCM used for both medicinal and dietary purposes, the present study provides a novel perspective on the utilization of traditional Chinese medicine as an herbal remedy for both medicinal and dietary purposes in the treatment of UC, thereby contributing to the development of effective therapeutic components.

## Data Availability

The data presented in this study are available in Supplementary Materials.

## Supplementary Materials

The following supporting information can be downloaded at: https://www.mdpi.com/article/10.3390/molecules29092154/s1, Figure S1. Base peak chromatograms of PCE in positive (A) and negative (B) mode. Figure S2. The original unadjusted and uncropped Western blot images. Table S1 Characterization of compounds identified from *Poria cocos* by UPLC-Q-Exactive-MS. Table S2. The standard curve of glucose was established by anthrone–vitriol method. Table S3. The quantification of the content of PC polysaccharides. Table S4. Primer sequence for qPCR.
