# Supplementary material for: Poria cocos Attenuated DSS-Induced Ulcerative Colitis via NF-κB Signaling Pathway and Regulating Gut Microbiota
Source: Molecules. 2024 May 6;29(9):2154. doi: 10.3390/molecules29092154 (PMC11085930; doi:10.3390/molecules29092154)
Supplement: Supplementary file 1 [file molecules-29-02154-s001.zip › molecules-2957401-supplementary.pdf]

Poria cocos attenuated DSS-induced ulcerative colitis via NF-κB signaling pathway and regulating gut microbiota

Xiaojun Song [1,†], Wei Wang [1,†], Yuanxiang Zhang [1], Li Liu [1], Zitong Zhao [1], Xuebin Shen [1], Lingyun Zhou [1], Yuanxiang Zhang [1,*], Daiyin Peng [2, 3,4,*] and Sihui Nian [1,5,6,7,*]

[1] School of Pharmacy, Wannan Medical College, Wuhu 241002, China; 20209071@stu.wnmc.edu.cn (W.W.); 20210027@wnmc.edu.cn (Y.Z.); liulilili@stu.wnmc.edu.cn (L.L.); 20209092@stu.wnmc.edu.cn (Z.Z.); sxb-chn@163.com (X.S.); zly4321@sina.com (L.Z.); tsongxj@wnmc.edu.cn (X.S.); niansihui@126.com (S.N.)

[2] School of Pharmacy, Anhui University of Chinese Medicine, Hefei 230012, China;

[3] Anhui Province Key Laboratory of Chinese Medicinal Formula, Hefei 230012, China;

[4] Xin'an Medicine, Key Laboratory of Chinese Ministry of Education, Anhui University of Chinese Medicine, Hefei 230038, China; pengdaiyin@163.com (D.P.)

[5] Anhui Provincial Engineering Laboratory for Screening and Re-evaluation of Active Compounds of Herbal Medicines in Southern Anhui, Wannan Medical College, Wuhu 241002, China; tsongxj@wnmc.edu.cn (X.S.); niansihui@126.com (S.N.)

[6] Institute of Modern Chinese Medicine, Wannan Medical College, Wuhu 241002, China; niansihui@126.com (S.N.)

[7] Center for Xin'an Medicine and Modernization of Traditional Chinese Medicine of IHM, Wannan Medical College, Wuhu 241002, China; niansihui@126.com (S.N.)

* Correspondence: 20210027@wnmc.edu.cn (Y.Z.); pengdaiyin@163.com (D.P.); niansihui@126.com (S.N.)

† These authors contributed equally to this work.

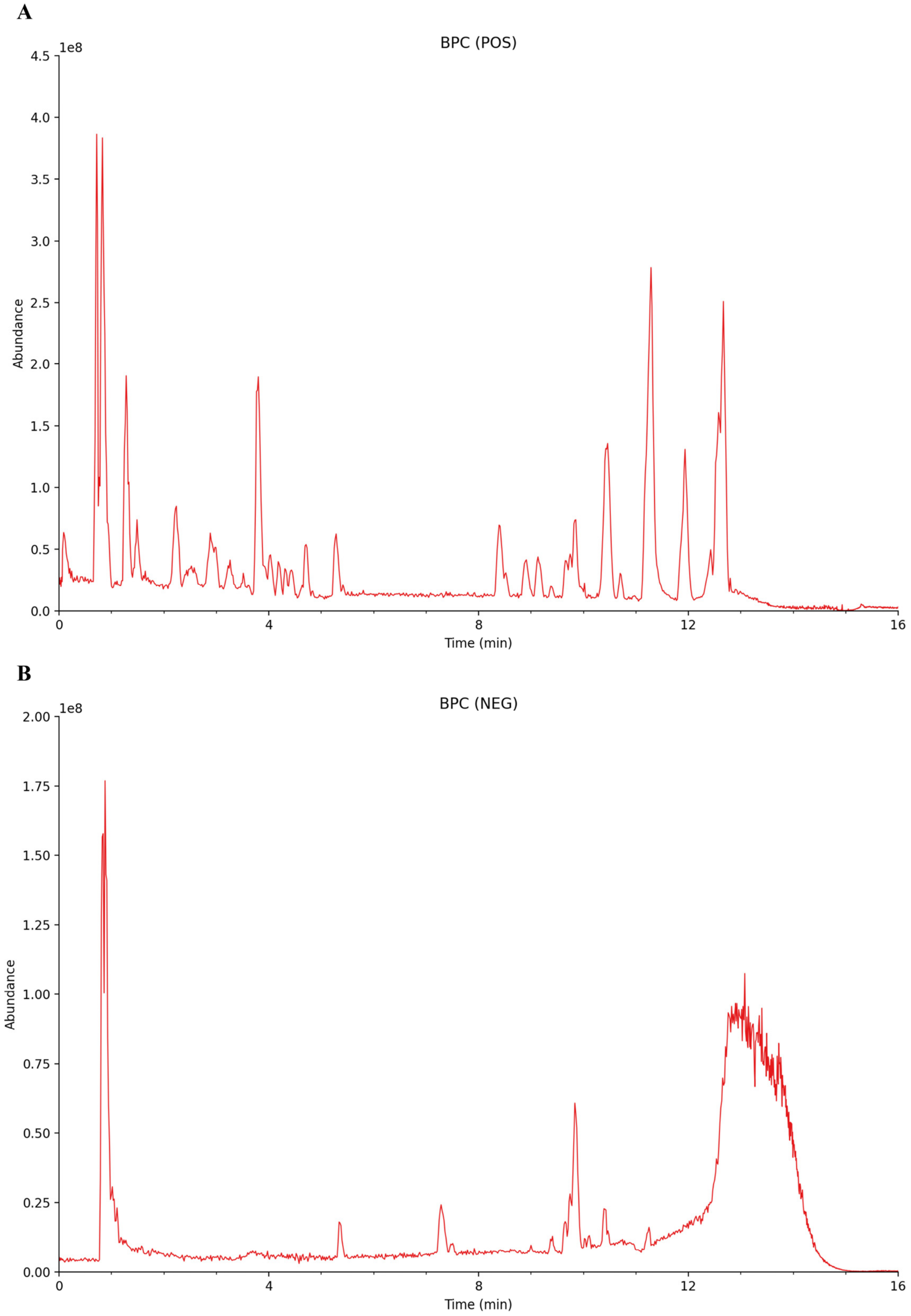

Figure S1. Base peak chromatograms of PCE in positive (**A**) and negative (**B**) mode.

A. The expression of Claudin-1 was assessed

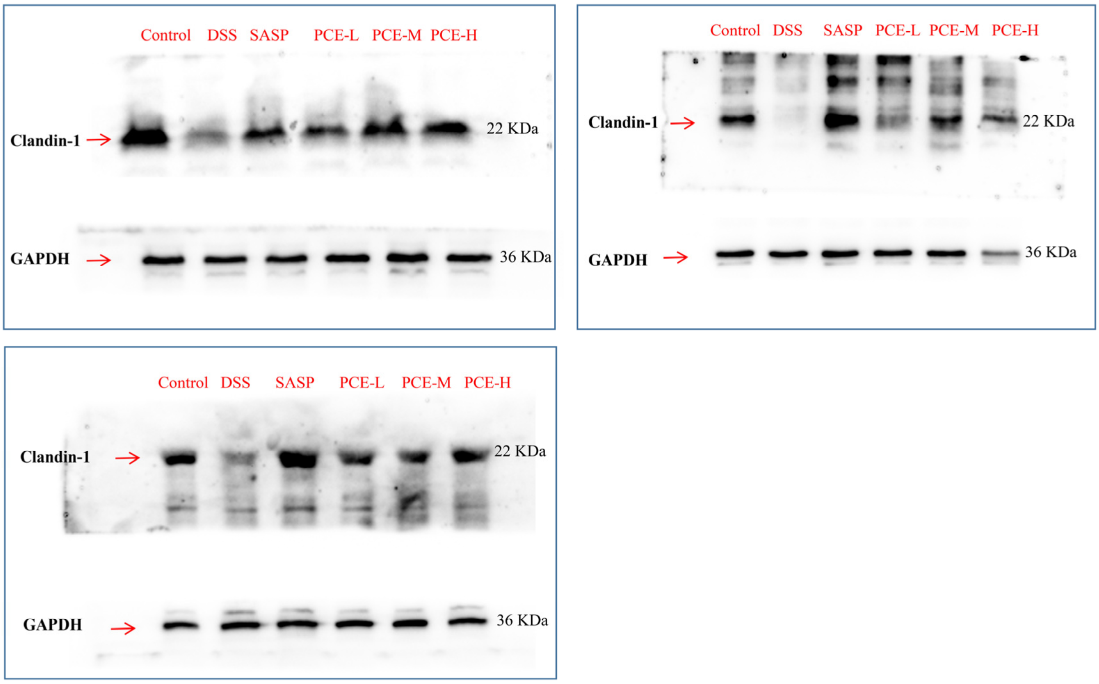

B. The expression of ZO-1 was assessed

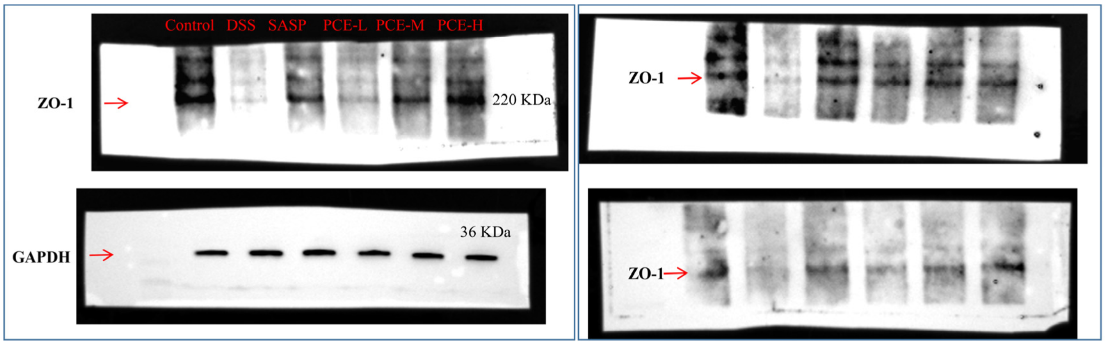

C. The expression of P-IκBα /IκBα was assessed

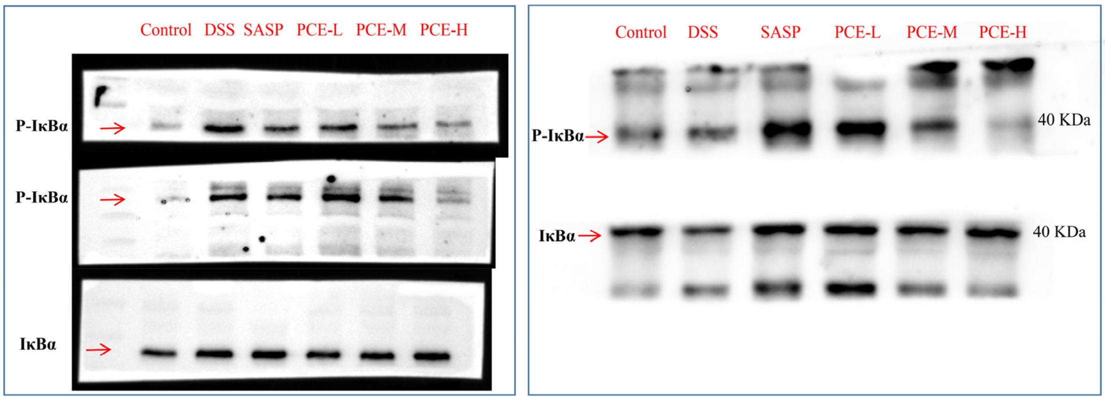

D. The expression of NF-κB p65 was assessed

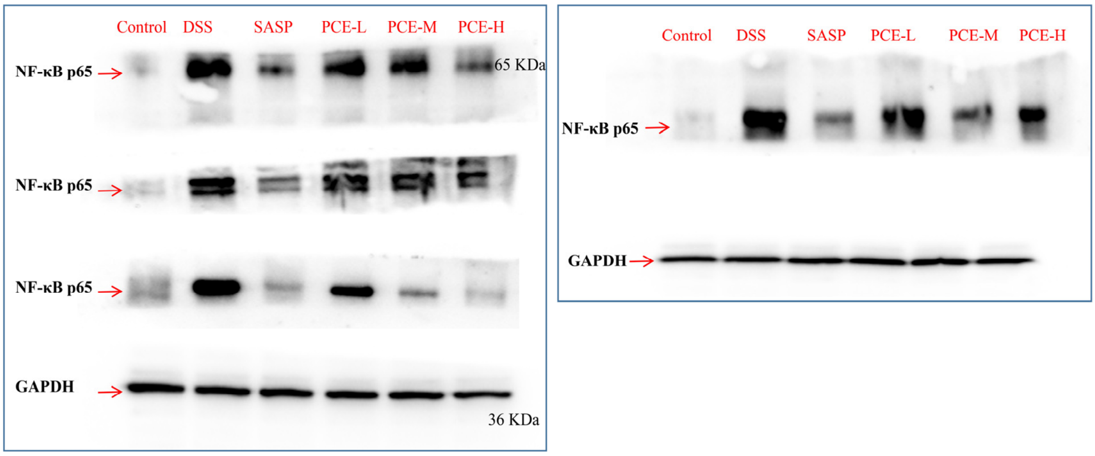

Figure S2. The original unadjusted and uncropped western blot images

Table S1 Characterization of compounds identified from *Poria cocos* by UPLC-Q-Exactive-MS.

No.	T_R (min)	Selected ion	Measured mass	Error (ppm)	Formula	Fragmentations (*m*/*z*)	Identified compounds	Identification level (A and B)	Type of compounds
1	0.73	$[M+NH_4]^+$	133.0974	1.74	$C_5H_9NO_2$	60.0564, 70.0658, 72.0815, 73.0848, 74.0244, 88.0398, 116.0708	L-Proline	A	Amino acid
2	0.77	$[M+H]^+$	175.1192	1.17	$C_6H_{14}N_4O_2$	112.0872, 116.0708, 130.0975, 137.5001, 140.0027, 149.0080, 149.5085, 157.1082, 175.1189	L-Arginine	A	Amino acid
3	0.8	$[M+H]^+$	156.0769	1.14	$C_6H_9N_3O_2$	95.0608, 110.0715, 156.0766	L-Histidine	A	Amino acid
4	0.83	$[M+H]^+$	120.0659	2.81	$C_4H_9NO_3$	56.0503, 74.0607, 102.0552, 120.0655	L-Threonine	A	Amino acid
5	0.83	$[M+H]^+$	166.0534	0.97	$C_5H_{11}NO_3S$	56.0503, 74.0243, 102.0553, 149.0265, 166.0527	Methionine sulfoxide	A	Amino acid
6	0.84	$[M-H]^-$	181.072	1.05	$C_6H_{14}O_6$	71.0139, 73.0296, 85.0296, 89.0245, 101.0245, 113.0245, 119.0350, 131.0350, 163.0614, 181.0719	Allitol	A	Carbohydrates
7	0.84	$[M+H]^+$	134.045	1.52	$C_4H_7NO_4$	57.0342, 60.0451, 69.0342, 70.0658, 74.0243, 88.0397, 116.0344, 134.045	L-Aspartic acid	A	Amino Acids
8	0.84	$[M-H]^-$	151.0614	1.18	$C_5H_{12}O_5$	85.0295, 87.0088, 89.0245, 101.0244, 113.0244, 119.0350, 131.0351, 133.0507, 151.0400, 151.0612	Arabinitol	A	Carbohydrates
9	0.86	$[M+H]^+$	153.0759	1.01	$C_5H_{12}O_5$	71.0498, 73.0291, 99.0444, 117.0548, 152.0567, 152.0699, 153.0404	Ribitol	A	Carbohydrates
10	0.87	$[M+H]^+$	222.0974	0.76	$C_8H_{15}NO_6$	126.0550, 134.0273, 134.0446, 138.0548, 144.0654, 168.0653, 186.0759, 204.0864, 222.0965	N-Acetyl-D-glucosamine	A	Carbohydrates
11	0.93	$[M-H]^+$	191.02	1.19	$C_6H_8O_7$	72.9931, 85.0295, 87.0088, 111.0088, 129.0195, 191.02	Citric acid	A	Organic acids

12	0.93	$[M+FA-H]^-$	289.0677	-0.3	$C_9H_{12}N_2O_6$	110.0248, 111.0202, 128.0352, 152.0354, 200.0562, 243.0617	Uridine	A	Nucleotides
13	0.93	$[M-H]^-$	323.0285	-0.26	$C_9H_{13}N_2O_9P$	78.9591, 96.9601, 96.9696, 111.0201, 124.9914, 138.9801, 211.0017, 323.0278	Uridine 5'-monophosphate	A	Nucleotides
14	0.93	$[M-H]^-$	177.0407	1.31	$C_6H_{10}O_6$	101.0246, 113.0254, 117.0194, 129.0197, 133.0298, 147.0304, 158.9256, 176.9364, 177.0187, 177.0412	1,4-D-Gulonolactone	A	Carbohydrates
15	1.21	$[M+H]^+$	130.0501	1.94	$C_5H_7NO_3$	70.0658, 84.0449, 84.0813, 87.0045, 113.9638, 129.0182, 130.0499	Pyroglutamic acid	A	Amino acid
16	1.28	$[M+H-H_2O]^+$	145.0497	0.88	$C_6H_{10}O_5$	71.0499, 81.0341, 85.0290, 86.0606, 98.0605, 99.0444, 100.0761, 127.0391, 144.0374, 145.0493	2-Hydroxyadipic acid	A	Fatty acid
17	1.28	$[M+H]^+$	268.1041	0.4	$C_{10}H_{13}N_5O_4$	136.0617, 268.1035	Adenosine	A	Nucleotides
18	1.37	$[M+H]^+$	284.099	0.22	$C_{10}H_{13}N_5O_5$	152.0566	Guanosine	A	Nucleotides
19	1.39	$[M+H]^+$	260.197	0.66	$C_{12}H_{25}N_3O_3$	101.1077, 129.1023, 130.0499, 147.0763, 147.1127, 148.0603, 243.1703, 259.1305, 260.1601, 260.1968	Lysylleucine	A	Peptide
20	1.49	$[M+H]^+$	132.1021	1.52	$C_6H_{13}NO_2$	69.0342, 69.0705, 86.0605, 86.0969, 87.0445, 97.0288, 115.0755	L-Leucine	A	Amino acid
21	2.24	$[M+H]^+$	328.1392	0.22	$C_{15}H_{21}NO_7$	120.081, 132.0808, 166.0862, 178.086, 264.1233, 292.1176, 310.1282, 328.1392	N-(1-Deoxy-1-fructosyl) phenylalanine	A	Amino acid
22	2.61	$[M+H]^+$	246.145	0.65	$C_{10}H_{19}N_3O_4$	200.1395, 201.1237, 211.1080, 212.0913, 228.1342, 229.1183, 245.0993, 246.0739, 246.1119, 246.1390	Asparaginylisoleucine	A	Peptide
23	2.81	$[M+H]^+$	281.1497	0.34	$C_{14}H_{20}N_2O_4$	55.0551, 72.0815, 120.0809, 136.0613, 136.0758, 149.0235, 182.0811, 281.1491	Valyltyrosine	A	Peptide

24	2.88	$[M+H]^+$	189.1235	0.64	$C_8H_{16}N_2O_3$	86.0969, 97.1017, 125.0963, 132.1019, 143.1177, 189.1016, 189.125	Glycyl-Isoleucine	A	Peptide
25	3.6	$[M+H]^+$	253.1186	1.09	$C_{12}H_{16}N_2O_4$	60.0452, 70.0658, 86.0969, 120.081, 166.0862, 207.1126, 235.1072, 253.1185	Serylphenylalanine	A	Peptide
26	3.71	$[M+H]^+$	231.1706	1.28	$C_{11}H^{22}N_2O_3$	70.0658, 72.0814, 86.0969, 185.1655, 231.1705	Isoleucyl-Valine	A	Peptide
27	3.93	$[M+H]^+$	231.1707	1.68	$C_{11}H_{22}N_2O_3$	72.0814, 132.1019, 231.17	Valylleucine	A	Peptide
28	4.23	[M+H]+	265.155	1.34	C14H20N_2O_3	55.0551, 72.0814, 102.0553, 120.0809, 146.06, 166.0857, 265.1544	Valylphenylalanine	A	Peptide
29	5.82	$[M+H]^+$	595.2018	-0.52	$C_{28}H_{34}O_{14}$	161.0594, 171.0284, 195.0291, 263.0549, 287.0910, 329.1022, 397.1285, 415.1377, 433.1492, 449.1445, 595.2018	Didymin	A	Flavonoid
30	6.01	$[M-H]^-$	201.1134	0.96	$C_{10}H_{18}O_4$	132.0564, 139.1128, 156.1026, 157.1232, 175.0625, 183.1024, 201.113	Sebacic acid	A	Fatty acid
31	6.75	$[M-H]^-$	215.1289	-0.04	$C_{11}H_{20}O_4$	153.1283, 197.1183, 215.0098, 215.1284	Undecanedioic acid	A	Fatty acid
32	6.94	$[M+H-H_2O]^+$	459.3101	-0.74	$C_{28}H_{44}O_6$	459.3101	Polyporusterone B	B	Steroid
33	7.4	$[M+H-H_2O]^+$	443.279	-0.35	$C_{27}H_{40}O_6$	293.2263, 311.2364, 375.2116, 407.2573, 425.2682, 443.2793	Lucidenic acid N	A	Triterpene
34	9.43	$[M+H]^+$	501.3574	-0.08	$C_{31}H_{48}O_5$	109.1016, 309.2211, 437.3416, 465.3379, 483.3476, 501.3578	29-hydroxydehydrotumulosic acid	B	Triterpene
35	9.78	$[M+H-H_2O]^+$	471.347	0.19	$C_{30}H_{48}O_5$	173.1325, 187.1481, 295.2414, 311.2357, 313.2524, 411.2519, 435.3256, 453.3361, 471.3466	Caulophyllogenin	A	Triterpene

36	9.84	[M+H-H2O]+	481.3311	-0.29	$C_{31}H_{46}O_5$	221.1324, 223.1476, 267.1750, 307.2043, 309.2208, 325.2166, 417.3165, 445.3123, 463.3246, 481.3364	6α-Hydroxypolyporenic acid C	B	Triterpene
37	9.96	[M+Na]+	537.3202	3.04	$C_{31}H_{46}O_6$	431.2968, 459.2907, 477.2989, 491.3156, 519.3114, 537.3225	Poricoic acid D	B	Triterpene
38	10.04	[M+FA-H]-	545.3488	0.82	$C_{31}H_{48}O_5$	59.0139, 75.0087, 85.0659, 429.2661, 447.2751, 499.3433, 545.3464	25-hydroxy-3-epidehydrotumulosic acid	B	Triterpene
39	10.33	[M+H]+	469.3316	0.79	$C_{30}H_{46}O_5$	95.0859, 223.148, 293.226, 311.2366, 433.3099, 451.3201, 469.3315	Quillaic acid	A	Triterpene
40	10.63	[M+H-H2O]+	467.3158	0.42	$C_{30}H_{44}O_5$	293.2257, 307.2055, 325.2158, 353.2476, 407.2555, 431.2941, 449.3035, 449.3430, 466.3396, 467.3143	Poricoic acid B	A	Triterpene
41	10.88	[M+H]+	499.3418	0.01	$C_{31}H_{46}O_5$	307.2060, 309.2206, 313.2752, 325.2152, 439.3222, 445.3099, 463.3225, 481.3320, 499.3358, 499.3483	Poricoic acid A	A	Triterpene
42	10.89	[M-H]-	497.3272	-0.04	$C_{31}H_{46}O_5$	423.2887, 497.3271	Poricoic acid BM	B	Triterpene
43	10.94	[M+H]+	487.342	0.43	$C_{30}H_{46}O_5$	173.1325, 185.1325, 309.2209, 327.2317, 433.3105, 451.3194, 468.3546, 469.3230, 487.3452, 487.3509	Poricoic acid G	B	Triterpene
44	11.12	[M+H]+	485.363	0.95	$C_{31}H_{48}O_4$	159.1170, 293.2261, 311.2362, 449.2987, 449.3427, 466.3399, 467.3058, 467.3500, 484.3590, 485.3661	Dehydrotumulosic acid	A	Triterpene
45	11.2	[M+H]+	487.3789	1.51	$C_{31}H_{50}O_4$	295.2427, 451.3553, 469.3652, 487.3761	Tumulosic acid	B	Triterpene
46	11.22	[M+H]+	483.3468	-0.12	$C_{31}H_{46}O_4$	223.1479, 309.2206, 447.3298, 465.3406, 483.3468	Polyporenic acid C	A	Triterpene
47	11.23	[M+H]+	543.368	-0.03	$C_{33}H_{50}O_6$	277.1786, 293.2258, 295.2418, 353.2473, 447.3271, 465.3362, 483.3495, 507.3467, 525.3582, 543.3691	29-hydroxydehydropachymic acid	B	Triterpene
48	11.48	[M+FA-H]-	587.3952	-0.23	$C_{35}H_{56}O_7$	541.3528, 587.3963	6α-Hydroxy-dehydropachymic acid	B	Triterpene

49	11.72	$[M-H]^-$	511.3428	-0.22	$C_{32}H_{48}O_5$	511.3428	Poricoic acid AM	B	Triterpene
50	11.87	$[M-H]^-$	525.3586	0.09	$C_{33}H_{50}O_5$	59.0139, 525.3583	Poricoic acid AE	B	Triterpene
51	12.07	$[M-H]^-$	513.3584	-0.29	$C_{32}H_{50}O_5$	513.3584	3-O-Acetyl-16α-hydroxytrametenolic acid	B	Triterpene
52	12.4	$[M-H]^-$	527.3742	-0.01	$C_{33}H_{52}O_5$	527.3742	Pachymic acid	B	Triterpene

* A denotes the compound was identified using a pure standard, while B indicates putative identification based on data from an online database.

Table S2. The standard curve of glucose was established by anthrone-vitriol method

**1%Glucose solution/ml**	0	0.2	0.4	0.6	0.8
**Water/ml**	1.0	0.8	0.6	0.4	0.2
**Absorbance value**	0	0.7583	1.4942	2.1136	2.8063
**Standard curve**	y = 3.5606x + 0.0256 (R^2 = 0.9989)				

Table S3. The quantification of the content of PC polysaccharides.

**Group**	**1**	**2**	**3**	**4**	**5**	**6**	**7**	**8**
**Absorbance value**	1.2416	1.1885	1.1094	1.0577	1.2462	1.1444	1.1105	1.2288
**Average value**	1.1659							
**Content of polysaccharide**	80.06%							

Table S4. Primer sequence for qPCR.

**Gene name**	**Forward Primer**	**Reverse Primer**
TNF-α	ACCCTCACACTCAGATCATCTTC	TGGTGGTTTGCTACGACGT
IL-6	CCAGTTGCCTTCTTGGGACT	CTGGTCTGTTGTGGGTGGTA
IL-1β	AGCAACAACATAAGCGTCAT	CCTCAAACTTGGCAATACTC
GAPDH	GAAGGTCGGTGTGAACGGAT	CCCATTTGATGTTAGCGGGAT
